# Recombination rate and protein evolution in yeast

**DOI:** 10.1186/1471-2148-7-235

**Published:** 2007-11-27

**Authors:** Tim Connallon, L Lacey Knowles

**Affiliations:** 1 Department of Ecology and Evolutionary Biology,Museum of Zoology, University of Michigan, Ann Arbor, MI 48109-1079, USA

## Abstract

**Background:**

Theory and artificial selection experiments show that recombination can promote adaptation by enhancing the efficacy of natural selection, but the extent to which recombination affects levels of adaptation across the genome is still an open question. Because patterns of molecular evolution reflect long-term processes of mutation and selection in nature, interactions between recombination rate and genetic differentiation between species can be used to test the benefits of recombination. However, this approach faces a major difficulty: different evolutionary processes (i.e. negative versus positive selection) produce opposing relationships between recombination rate and genetic divergence, and obscure patterns predicted by individual benefits of recombination.

**Results:**

We use a combination of polymorphism and genomic data from the yeast *Saccharomyces cerevisiae *to infer the relative importance of nearly-neutral (i.e. slightly deleterious) evolution in different gene categories. For genes with high opportunities for slightly deleterious substitution, recombination substantially reduces the rate of molecular evolution, whereas divergence in genes with little opportunity for slightly deleterious substitution is not strongly affected by recombination.

**Conclusion:**

These patterns indicate that adaptation throughout the genome can be strongly influenced by each gene's recombinational environment, and suggest substantial long-term fitness benefits of enhanced purifying selection associated with sexual recombination.

## Background

Genetic drift is expected to overpower natural selection when selection is weak and effective population size (*N*_*e*_) is small [1-3]. Recombination increases the effective population size in which genes evolve by reducing interference between linked loci under selection [4,5]. As a result, recombination is expected to facilitate the spread of beneficial mutations and the elimination of deleterious mutations [6,7]. Because recombination rates vary between different regions of a genome [e.g. yeast: [8]; *Drosophila*: [9]; Mammals: [10]; plants: [11]], adaptation at the molecular level might be strongly affected by each gene's recombinational environment – genes evolving in low recombination regions are expected to be poorly adapted relative to those in high recombination regions [12,13].

Comparative genome analyses are potentially useful for assessing whether recombination promotes adaptation because long-term evolutionary processes are reflected in patterns of genetic divergence between species [13-19]. However, genomic approaches face a major challenge – multiple processes can contribute to evolutionary divergence between species and each predicts a different relationship between protein evolution and recombination rate (Table 1). The rate of neutral substitution is unaffected by recombination and will tend to reduce correlations between recombination rate and total nucleotide divergence between species [20], mildly deleterious (i.e. nearly neutral) substitutions will generate a negative correlation between recombination and divergence [21], and adaptive substitutions generate a positive correlation between recombination and divergence [22].

**Table 1:** Different processes of molecular evolution produce different correlations between recombination rate and nucleotide divergence between species^1^.

**Model of evolution**	**Fitness effect of substitutions**^2^	**Correlation between recombination rate and divergence**
Neutral	*s *= 0	None
Slightly deleterious	-1/(2*N*_*e*_) <*s *< 0	Negative
Adaptive	*s *> 0	Positive

^1 ^See text for details

^2 ^The selection coefficient, *s *(-1 ≤ *s *< ∞), reflects the strength and direction of selection acting upon new mutations during the process of fixation

The relationship between recombination and divergence will be shaped by the predominant process of molecular evolution (i.e. neutral; slightly deleterious; adaptive). To test whether recombination facilitates adaptation throughout the genome (by enhancing purifying and positive selection), genes evolving under purifying selection and those evolving via positive selection should be analyzed separately, as each predicts a different relationship between divergence and recombination rate. Unfortunately, inferring the processes causing molecular divergence has traditionally been problematic without detailed within- and between-species genetic data [23], which limits the extent of the genome that can be analyzed.

**Figure 1:**
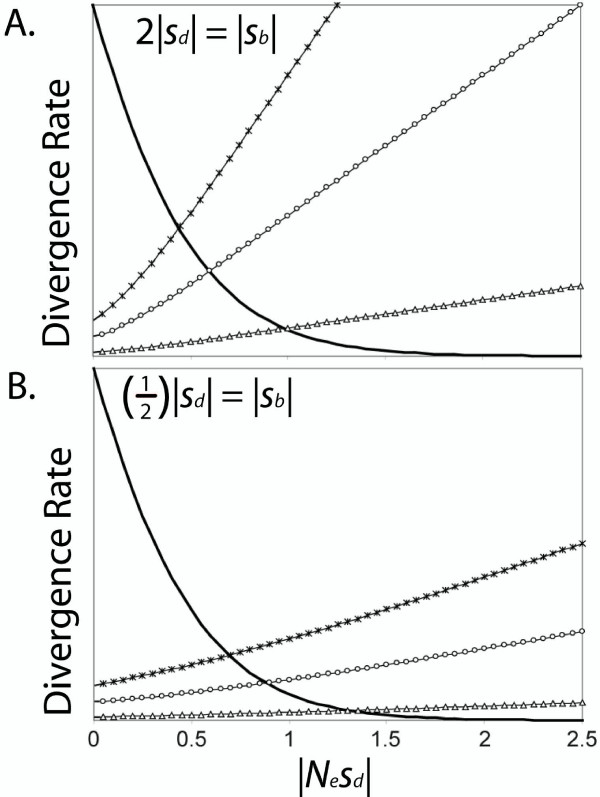
Substitution rate as a function of the effective strength of selection. The bold solid curve represents the slightly deleterious divergence rate between species. Remaining curves represent adaptive divergence under three scenarios: open triangles when the beneficial mutation rate (*u*_*b*_) is 1% of the deleterious mutation rate (*u*_*d*_); open circles when *u*_*b *_is 5% of *u*_*d*_; 'X-marks' when *u*_*b *_is 10% of *u*_*d*_. The strength of selection against deleterious mutations (|*N*_*e*_*s*_*d*_|) is shown on the x-axis, where *s*_*d *_is the average strength of purifying selection. A. The average strength of positive selection (*s*_*b*_) is equal to the average strength of purifying selection (*s*_*d*_); B. *s*_*b *_is twice as strong as *s*_*d*_; C. *s*_*d *_is twice as strong as *s*_*b*_. When selection is weak, mildly-deleterious substitutions outnumber adaptive substitutions; divergence in weakly-selected genes is therefore predicted to be negatively correlated with local recombination rate. As the strength of selection increases, adaptive substitutions predominate; divergence in strongly-selected genes is therefore predicted to be positively correlated with recombination rate. The adaptive divergence rate is *u*_*b*_(1 - *e*^-4*Nsp*^)/(1 - *e*^-4*Ns*^), where *s *is the average benefit conferred by each mutation, and *p *is the initial frequency of each mutation (results for *p *= 0.0001 are shown). The slightly deleterious rate is *u*_*d*_(1 - *e*^-4*Nsp*^)/(1 - *e*^-4*Ns*^), where *u*_*d *_is the deleterious mutation rate, and *s *is the average cost of each mutation (equations modified from [30]; *N*_*e *_= *N*). The assumption that the beneficial mutation rate is much smaller than the deleterious mutation rate is supported by theory and mutation accumulation experiments [38,55-57; but see 58,59].

Here we take an alternative approach. By capitalizing on an extensive volume of yeast (*Saccharomyces *spp.) genomic and polymorphism data, individual genes can be partitioned by their 'opportunity' for slightly deleterious evolution, and benefits of recombination can be tested. Furthermore, direct estimates of local recombination rates are available for most genes in the *S. cerevisiae *genome, whole-genome sequencing projects provide data for estimating protein evolutionary rates, and previous studies have revealed that the average strength of selection predictably varies between genes with different functional attributes [reviewed in [22]; see below]. Evolutionary theory predicts that mildly deleterious substitutions will accumulate readily in genes subject to weak selection, but not in those subject to strong selection (see Fig. 1). To the extent that substitutions differentiating species are often deleterious (e.g., genes with weakly-selected mutations; Fig. 1 – small |*N*_*e*_*s*_*d*_|), increased recombination is expected to decrease the rate of protein evolution by inhibiting the spread of deleterious mutations. However, in genes with little opportunity for slightly deleterious divergence (e.g. genes subject to strong purifying selection; Fig. 1 – large |*N*_*e*_*s*_*d*_|), recombination will increase the rate of divergence by enhancing the spread of beneficial mutations (to the extent that such mutations frequently arise).

## Results & Discussion

### Inferring the fitness effects of deleterious mutations

Highly expressed genes appear to evolve under stronger purifying selection than low-expressed genes (although the mechanistic basis of this pattern is still debated; [24-27]). Consequently, gene expression level is a good predictor of the average fitness effect of deleterious mutations. Experimental gene knockouts have also identified suites of genes that are essential for survival, while many others are nonessential. To the extent that whole gene knockout phenotypes reflect the fitness effects of individual mutations, mutations in essential genes are predicted to have larger fitness effects than mutations in nonessential genes [25,28-30]. Lastly, proteins have variable numbers of interaction partners (protein-protein interactions per gene – PPI – range up to nearly 300 PPI in yeast; Connallon & Knowles unpub.), which indicate the level of constraint due to pleiotropy [25]. Because individual mutations are likely to disrupt more cellular processes in genes with many PPI compared to those with few PPI, purifying selection is expected to be stronger in genes with many PPI [31].

Previous inferences of the strength of purifying selection acting on different gene categories are based on an observed elevated rate of nonsynonymous substitution in low-expressed nonessential genes with few PPI [25], which assumes that elevated rates of substitution are caused by genetic drift. An alternative possibility is that rapidly evolving genes undergo frequent bouts of positive selection. To test this assumption, we analyzed available polymorphism data from *S. cerevisiae *genes (see Methods and Additional file 1). Under a neutral/nearly-neutral model, patterns of within species polymorphism are expected to mirror patterns of interspecific substitution (i.e. genes with high substitutions rates also exhibit high levels of polymorphism [32]). Positive selection decouples patterns of polymorphism and divergence and is expected to increase the number of substitutions relative to polymorphisms [33].

**Figure 2:**
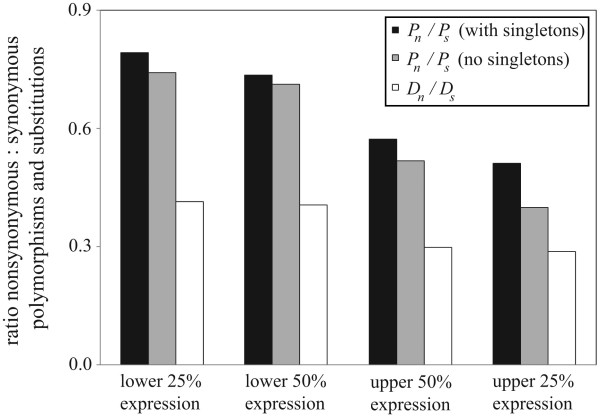
Ratios of replacement to silent polymorphism (*P*_*n*_*/P*_*s*_) in *S. cerevisiae*, and substitutions (*D*_*n*_*/D*_*s*_) between *S. cerevisiae *and *S. paradoxus*. Results were obtained by pooling polymorphism and divergence data for multiple genes within each expression category (see Fig. S1 for similar results using a different approach). *P*_*n*_/*P*_*s *_ratios are lower in highly expressed genes: upper vs. lower 50% with singletons, *P *= 0.172; upper vs. lower 25% with singletons, *P *= 0.026; upper vs. lower 50% without singletons, *P *= 0.203; upper vs. lower 25% without singletons, *P *= 0.023. *D*_*n*_/*D*_*s *_ratios are lower for highly expressed genes for all comparisons (*P *< 0.0001).

Low-expressed genes harbor more nonsynonymous polymorphisms (represented by the *P*_*n*_/*P*_*s *_ratio; *P*_*n *_and *P*_*s *_refer to nonsynonymous to synonymous polymorphisms, respectively) and substitutions (*D*_*n*_/*D*_*s*_), than highly expressed genes, consistent with the neutral/nearly-neutral model (Fig. 2; see Additional file 1). Low-expressed genes also harbor higher levels of moderate- to high-frequency polymorphism (i.e. "non-singleton" polymorphism). High frequency polymorphisms are not expected to be strongly deleterious (see [34]), but rather, will consist of neutral and slightly deleterious mutations – mutations that can potentially become fixed via genetic drift. *D*_*n*_/*D*_*s *_ratios are significantly lower than *P*_*n*_/*P*_*s *_ratios for all gene expression categories (G test; singletons included: *P *< 0.0001; singletons excluded: P < 0.005, except for upper 25% expressed genes: *P *> 0.1), indicating that the predominant pattern of selection on yeast genes is purifying. Similar results are reached by partitioning the data into gene essentiality categories (see Additional file 1; a meaningful statistical analysis based on PPI was not possible due to small sample size). The results strongly support previous inferences of selection intensity based on divergence data, and suggest that nonessential genes with low expression are relatively likely to evolve under a nearly-neutral process.

### Recombination and protein divergence

**Figure 3:**
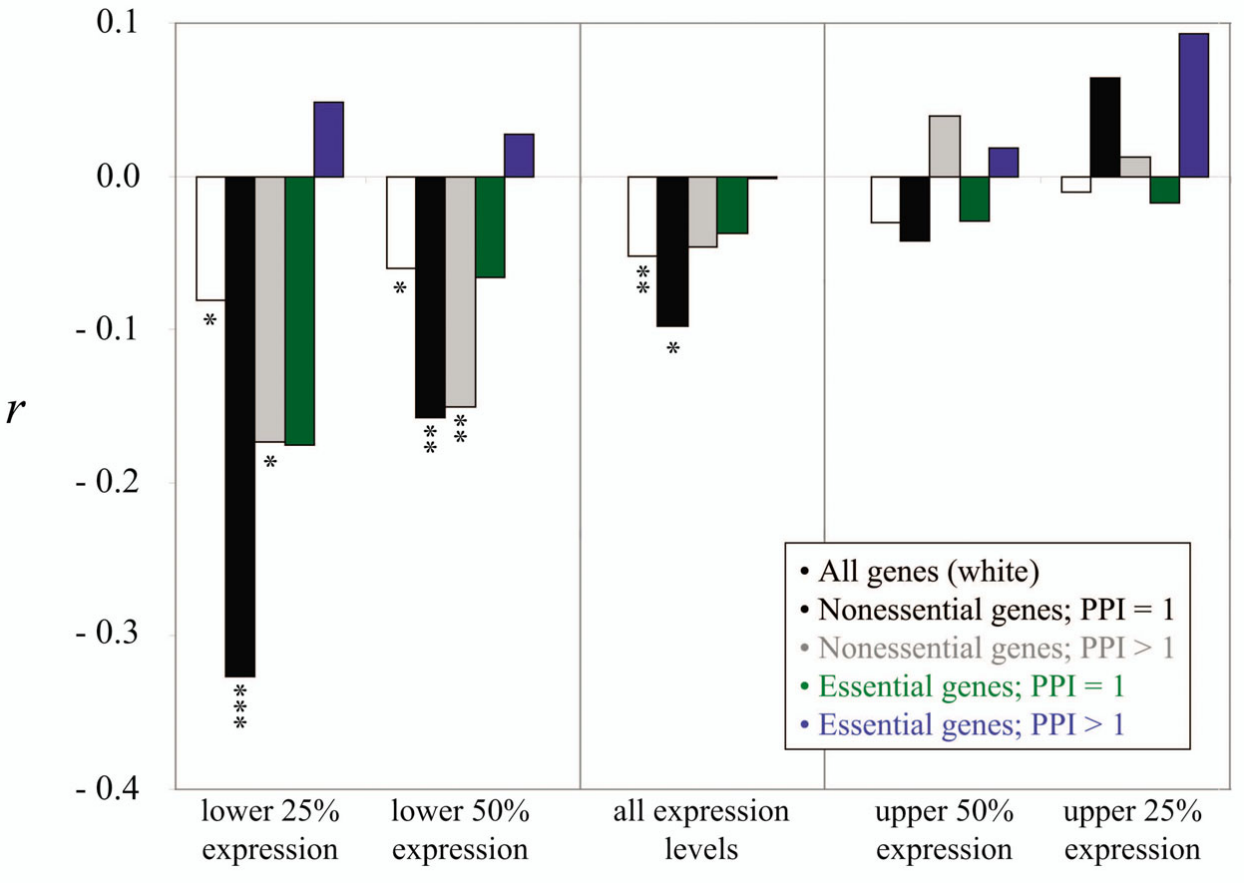
Recombination and protein evolutionary rate. The relationship (*r *= partial correlation coefficient; see materials and methods) between recombination rate and *dN *for five gene expression intervals: the lower gene expression quartile (2.25 to 3.15 log mRNA abundance), the lower 50% expression (3.31 to 4.54 log mRNA abundance), and the upper gene expression quartile (3.53 to 4.54 log mRNA abundance). * *P *< 0.05; ** *P *< 0.01; *** *P *< 0.001 (after Bonferroni correction for five comparisons).

Analysis of 4786 genes in the yeast species *Saccharomyces cerevisiae *and *S. paradoxus *shows that nonsynonymous divergence is weakly negatively correlated with recombination rate (partial *r *= -0.052, *P *< 0.01 after Bonferroni correction; as previously reported by Pal et al. [14]). This negative relationship is markedly stronger for low-expressed genes, particularly nonessential genes with few PPI (Fig. 3). In contrast, the divergence of highly expressed genes tends to correlate positively with recombination rate, though all such associations are weak. For both classes of nonessential genes as well as for the entire dataset, the relationship between recombination and divergence in low-expressed genes is more negative than that of highly expressed genes (Upper vs. lower 25% expression quartiles: all genes, *n*_*low *_= 1197, *n*_*high *_= 1197, *P *= 0.041; nonessential PPI = 1, *n*_*low *_= 246, *n*_*high *_= 218, *P *< 0.0001; nonessential PPI > 1, *n*_*low *_= 222, *n*_*high *_= 142, *P *= 0.041. Upper vs. lower 50% expression quantiles: all genes, *n*_*low *_= 2393, *n*_*high *_= 2393, *P *= 0.148; nonessential PPI = 1, *n*_*low *_= 424, *n*_*high *_= 411, *P *= 0.046; nonessential PPI > 1, *n*_*low *_= 435, *n*_*high *_= 327, *P *= 0.005).

These results clearly show that recombination can influence the rate of protein evolution at a genome wide scale and that the impact of recombination rate variation is strongest for low-expressed, nonessential genes with few PPI. Associations between recombination and divergence rate cannot be explained by covariation between recombination rate and several variables that independently affect protein evolution (the effects were controlled for; see Methods). Estimates of the relative recombination rate between genes are coarse and limited by the quality of the *S. cerevisiae *recombination map, and there are potential evolutionary changes in recombination between *S. cerevisiae *and *S. paradoxus*. However, both of these factors will decrease the strength of associations between divergence and recombination, and will cause our test to be conservative.

Mutation bias is also unlikely to account for the effect of recombination on protein evolution. We present associations between recombination and divergence at nonsynonymous sites (*dN*) rather than between recombination and *dN/dS *ratios because synonymous sites are under selection in yeast [e.g. [35]]. Indeed, codon usage bias (*F*_*op*_) is positively correlated with recombination (as previously reported [35]), most strongly for highly expressed genes, which presumably have stronger selection for optimal codons (highest 50% expression partial *r*_*rec*-*Fop *_= 0.214 vs. lowest 50% *r*_*rec*-*Fop *_= 0.100; highest 25% expression partial *r*_*rec*-*Fop *_= 0.249 vs. lowest 25% *r*_*rec*-*Fop *_= 0.089; *P *< 0.0001 for both comparisons). As a consequence, *dS *is negatively correlated with recombination (highest 50% expression partial *r*_*rec*-*Fop *_= -0.131 vs. lowest 50% *r*_*rec*-*Fop *_= -0.077, *P *= 0.031; highest 25% expression partial *r*_*rec*-*Fop *_= -0.122 vs. lowest 25% *r*_*rec*-*Fop *_= -0.075, *P *= 0.123). Furthermore, direct estimates indicate higher mutation rates in regions of high recombination [36,37], which should make our tests conservative. Despite these caveats with respect to using *dS *to estimate underlying mutational dynamics across the genome, *dN*/*dS *produces nearly-identical patterns of covariation with recombination (see Additional file 1, Fig. S3).

The results are consistent with evolutionary theory suggesting that recombination enhances the efficacy of selection [e.g. [6,12]]. Mutations with weak fitness effects respond to selection when the effective population size (*N*_*e*_) is large, but evolve via genetic drift when *N*_*e *_is small. By increasing *N*_*e*_, recombination enhances the power of selection and minimizes genetic drift. Furthermore, the adaptive consequences of recombination may be extreme in yeast since most genes in the yeast genome can be defined by weak purifying selection (~75% of genes are nonessential; ~50% have one PPI; TC & LLK unpub.). Such genes also tend to reside in genomic regions with relatively low recombination frequencies (see Additional file 1, Fig. S4). The correlations revealed by the data are particularly striking when one considers the method by which the genes are partitioned. Functional genomic data permits classification of genes according to their relative opportunities for slightly deleterious evolution. However, multiple types of substitutions (i.e. slightly deleterious, neutral, and beneficial) are likely to contribute to each gene's total genetic divergence between species. This plurality should dampen patterns predicted by any single processes, and will cause the conclusions presented here to be conservative.

Why does highly-expressed gene divergence show no correlation with recombination rate? There are two major possibilities. If genes under stronger selection tend to experience fewer beneficial mutations, their overall divergence rate might be relatively unaffected by local recombination. This might occur because strongly-selected genes are closer to perfection than weakly-selected genes and therefore have less opportunity for improvement, or because tradeoffs via pleiotropy (as indicated by high PPI [31]) limit the opportunity for beneficial mutations [38-40]. Secondly, selection for beneficial mutations might be very strong. The adaptive impact of varying recombination rate (and thus *N*_*e*_) is expected to decrease with the strength of selection (i.e. *s*; [3,41]). If beneficial mutations tend to be strongly advantageous, they will tend to become fixed in low or in high recombinational environments.

## Conclusion

This study shows that recombination reduces evolutionary divergence in genes under relatively weak purifying selection (e.g. low-expressed, nonessential, few PPI), and at best, marginally increases divergence in genes under strong purifying selection (e.g. highly expressed, essential, many PPI). This pattern suggests that enhanced purifying selection is a primary long-term benefit of recombination in nature. The efficient removal of deleterious mutations might increase the competitive ability of sexual species and contribute to the observed ubiquity of sexual reproduction in eukaryotes [6,42].

While this interpretation is appealing and supported by both theory and data from other taxa [e.g. [12,13]], it should be noted that inferences about the processes driving nucleotide divergence between species are tentative and reflect a major limitation of molecular divergence data. Future studies using entire-genome polymorphism and divergence data can add resolution by estimating the proportion of adaptive substitutions per gene [see [23]]. Such estimates, combined with inferences about the slightly deleterious substitution rate (derived from expression, essentiality and PPI data), will permit a much improved analysis of the benefits of recombination.

## Methods

### Data

Publically available polymorphism data was obtained via [43] and [44]. Genes with at least four samples from *S. cerevisiae *and at least one polymorphic site were included in the analysis, resulting in a dataset of 35 genes (lower 25% expression, *n *= 11; lower 50% expression, *n *= 12; upper 50% expression, *n *= 23; upper 25% expression, *n *= 17; essential genes, *n *= 7; nonessential genes, *n *= 28), comprising 34443 nonsynonymous, and 9975 synonymous nucleotide sites. The mean number of samples per gene was n¯
 MathType@MTEF@5@5@+=feaafiart1ev1aaatCvAUfKttLearuWrP9MDH5MBPbIqV92AaeXatLxBI9gBaebbnrfifHhDYfgasaacPC6xNi=xH8viVGI8Gi=hEeeu0xXdbba9frFj0xb9qqpG0dXdb9aspeI8k8fiI+fsY=rqGqVepae9pg0db9vqaiVgFr0xfr=xfr=xc9adbaqaaeGacaGaaiaabeqaaeqabiWaaaGcbaGafmOBa4Mbaebaaaa@2D50@ = 22. Orthologous sequences from *S. paradoxus *were obtained via BLAST search at [45].

Per gene recombination rates for *S. cerevisiae *are those reported by Gerton et al. [8] [data available at [46]]. These estimates refer to recombination rates per sexual generation. The total, per generation recombination rate during the evolutionary history of each gene is the product of the rate under sexual reproduction (*R*_*sex*_) and the frequency of outcrossing (*O*_*c*_); *R*_*TOT *_= *R*_*sex*_*O*_*c *_(modified from [47]). Because the exact value of *O*_*c *_will be the same for all genes in the genome, *R*_*sex *_per gene *i*, relative to *R*_*sex *_for other genes, will be the same as *R*_*TOT *_per gene *i*, relative to *R*_*TOT *_for other genes (i.e.RTOT(i)RTOT(max⁡)=Rsex(i)OcRsex(max⁡)Oc=Rsex(i)Rsex(max⁡))
 MathType@MTEF@5@5@+=feaafiart1ev1aaatCvAUfKttLearuWrP9MDH5MBPbIqV92AaeXatLxBI9gBaebbnrfifHhDYfgasaacPC6xNi=xH8viVGI8Gi=hEeeu0xXdbba9frFj0xb9qqpG0dXdb9aspeI8k8fiI+fsY=rqGqVepae9pg0db9vqaiVgFr0xfr=xfr=xc9adbaqaaeGacaGaaiaabeqaaeqabiWaaaGcbaWaaeWaaeaacqWGPbqAcqGGUaGlcqWGLbqzcqGGUaGljuaGdaWcaaqaaiabdkfasnaaBaaabaGaemivaqLaem4ta8KaemivaqfabeaacqGGOaakcqWGPbqAcqGGPaqkaeaacqWGsbGudaWgaaqaaiabdsfaujabd+eapjabdsfaubqabaGaeiikaGIagiyBa0MaeiyyaeMaeiiEaGNaeiykaKcaaOGaeyypa0tcfa4aaSaaaeaacqWGsbGudaWgaaqaaiabdohaZjabdwgaLjabdIha4bqabaGaeiikaGIaemyAaKMaeiykaKIaem4ta80aaSbaaeaacqWGJbWyaeqaaaqaaiabdkfasnaaBaaabaGaem4CamNaemyzauMaemiEaGhabeaacqGGOaakcyGGTbqBcqGGHbqycqGG4baEcqGGPaqkcqWGpbWtdaWgaaqaaiabdogaJbqabaaaaOGaeyypa0tcfa4aaSaaaeaacqWGsbGudaWgaaqaaiabdohaZjabdwgaLjabdIha4bqabaGaeiikaGIaemyAaKMaeiykaKcabaGaemOuai1aaSbaaeaacqWGZbWCcqWGLbqzcqWG4baEaeqaaiabcIcaOiGbc2gaTjabcggaHjabcIha4jabcMcaPaaaaOGaayjkaiaawMcaaaaa@7585@, and *R*_*sex *_should accurately capture relative rates of total recombination for each gene. Furthermore, because 0 <*O*_*c *_< 1 [47], the critical transition between *R*_*TOT *_≈ 0 to *R*_*TOT *_> 0, predicted to most strongly impact the efficacy of selection [48], will be represented with yeast genes.

Protein divergence data for *S. cerevisiae *and *S. paradoxus *orthologous genes were kindly provided by D. Allan Drummond (see [24] for details). Gene expression values for *S. cerevisiae *were calculated from seven time period estimates during the diauxic shift by DeRisi et al. [49] [data available at [50]]. Average and maximum expression levels for the seven time periods were calculated using the method of Kliman et al. [35]. Results for average gene expression (*E*_*avg*_) across the time periods are presented here; results do not differ when maximum expression values (*E*_*max*_) are used. Essential genes were identified through the GeneMerge database [51]. Gene length, dispensability, protein-protein interactions and genome map positions were obtained from the *Saccharomyces *Genome Database. Genes with no known interaction partner were excluded from analyses involving PPI as a variable. Space between genes (SBG) was calculated by the method of Hey & Kliman [9]. Recombination, expression, length, SBG and divergence estimates were available for 4786 genes in total, including essential genes with two or more PPI (*n *= 329), essential genes with 1 PPI (*n *= 195), nonessential genes with two or more PPI (*n *= 762), and nonessential genes with 1 PPI (*n *= 835). Our dataset is available upon request.

### Analysis

Population samples for each gene were aligned with ClustalW [52], available online, and manually adjusted. *P*_*n*_, *P*_*s*_, *D*_*n*_, and *D*_*s *_values were calculated with DnaSP, Version 4.10 [53]. Watterson's estimate of silent nucleotide diversity (theta) was calculated by hand (as described in [54]). The complete polymorphism dataset is provided in Additional file 1.

Genes were classified into low and high expression level categories, based on quantile partitions. These correspond with ranges of lower 25%: 2.25 to 3.15 (*n *= 1197); lower 50%: 2.25 to 3.31 (*n *= 2393); upper 50%: 3.31 to 3.53 (*n *= 2393); and upper 25%: 3.53 to 4.54 (*n *= 1197) log mRNA abundance.

All divergence estimates were log_10 _transformed to facilitate linear comparisons, which are presented (values of *dN *= 0 were converted to *dN *= 0.0001 prior to log transformation); the results are robust and also obtained with nonparametric comparisons (TC & LLK unpub.). Partial correlation analysis was used to compare recombination rate with *dN *(the rate of nonsynonymous substitutions); the same results were obtained for the comparison between recombination rate and *dN/dS*. The partial *r *statistic reported here reflects the association between recombination rate and protein divergence after associations between gene expression, gene length, and SBG were removed. These factors are known to influence patterns of protein evolution [[25]; TC & LLK unpub.], are all correlated with one another (i.e. recombination is positively correlated with expression and gene density, but negatively correlated with length), and can therefore give rise to spurious correlations between the variables of interest. All statistical analyses were carried out with JMP (SAS Institute). Statistical comparisons between *r *for different gene categories were carried out with software available online . Bonferroni corrections for multiple comparisons (α/5; because of the 5 categories explored in Figure 2) were used to adjust *P *values of statistical significance.

## Authors' contributions

TC and LLK participated in designing of the study, analyzing the data and writing the manuscript. Both authors read and approved the final manuscript.

## Supplementary Material

Additional file 1 (Connallon&Kowles Supplementary Data): includes the entire polymorphism dataset, four supplementary figures (Figs. S1, S2, S3, S4), and additional details of the polymorphism analysis. Click here for file
