# Supplementary material for: Recombination rate and protein evolution in yeast
Source: BMC Evol Biol. 2007 Nov 27;7:235. doi: 10.1186/1471-2148-7-235 (PMC2211315; doi:10.1186/1471-2148-7-235)
Supplement: Additional file 1 — (Connallon&Kowles Supplementary Data): includes the entire polymorphism dataset, four supplementary figures (Figs. S1, S2, S3, S4), and additional details of the polymorphism analysis. [file 1471-2148-7-235-S1.pdf]

Supplementary Data (Connallon & Knowles, *BMC Evolutionary Biology*)

Contents

I. Inferring Processes of Selection Acting on Yeast Genes with Polymorphism Data

- Variables
- Models of Evolution
- Polymorphism and Divergence Data
- Additional Results
    - Fig. S1
    - Fig. S2

II. Additional Figures

- Fig. S3
- Fig. S4

III. Additional References

I. Inferring Processes of Selection Acting on Yeast Genes with Polymorphism Data

Here we provide additional details of the *S. cerevisiae* polymorphism dataset and the conceptual framework for inferring the processes and strength of selection acting upon yeast genes.

Variables

- D_n The number of amino acid replacement (nonsynonymous) substitutions per gene, between *S. cerevisiae* and *S. paradoxus.*
- D_s The number of silent (synonymous) substitutions per gene, between *S. cerevisiae* and *S. paradoxus.*
- P_n The number of segregating amino acid replacement polymorphisms per gene, within *S. cerevisiae.*
- P_s The number of segregating silent polymorphisms per gene, within *S. cerevisiae.*
- Theta Watterson's (1975) estimator of nucleotide variation, per nucleotide site

Models of Evolution (see McDonald & Kreitman 1991; Smith & Eyre-Walker 2002; Bierne & Eyre-Walker 2004)

- Under a neutral model ($s = 0$), D_n, D_s, P_n, and P_s are unfiltered by selection and are proportional to the mutation rate; $D_n/D_s = P_n/P_s$.
- Under a positive selection model, beneficial substitutions inflate D_n relative to D_s, but marginally contribute to standing genetic variation. Thus, D_n/D_s is expected to increase relative to P_n/P_s, as the number of adaptive substitutions increases.
- Under a purifying selection model, there are two possibilities:
  1. Purifying selection is strong. Deleterious mutations contribute to P_n but not to D_n; increased P_n is not associated with increased D_n. Because selection is strong, deleterious alleles remain at low frequency; thus $(D_n/D_s)/(P_n/P_s) < 1$, but only when low frequency polymorphism (i.e. "singleton") is included.
  2. Purifying selection is weak. Deleterious mutations contribute to P_n, and potentially to D_n; increased P_n is associated with increased D_n. Because selection is weak, deleterious alleles often reach moderate to high frequency; thus $(D_n/D_s)/(P_n/P_s) < 1$, whether low frequency polymorphism is included or not.

Polymorphism and Divergence Data

		With singletons		No singletons					
Gene	*N*	P_s	P_n	P_s	P_n	D_s	D_n	Expression Quantile[(1)]	Essential?
ACT1	10	1	0	1	0	14	0	4	yes
CCA1	73	11	1	4	1	53	10	2	yes
CDC19	29	3	0	3	0	22	6	4	yes
CWP1	15	7	5	6	1	57	29	4	no
FIG1	20	8	8	6	2	86	11	1	no

FZF1	29	6	4	6	4	87	85	1	no
GCN4	19	5	6	2	1	55	17	4	no
HHT2	9	4	0	4	0	15	0	4	no
HIS3	15	7	4	2	2	no BLAST hit		3	no
MBP1	9	7	4	1	2	69	12	4	yes
MKT1	4	1	2	1	2	185	52	4	no
MLH1	14	6	14	5	8	192	74	1	no
MLS1	77	12	4	6	2	65	7	4	no
NEW1	6	3	1	3	0	23	8	4	no
PDC1	6	0	4	0	1	34	11	4	no
PDR10	75	6	7	4	5	66	28	4	no
PEA2	17	18	14	7	5	111	35	1	no
PHD1	29	6	7	5	6	71	35	3	no
PMS1	5	3	1	0	0	214	117	1	no
RME1	13	3	4	0	2	65	68	4	no
RNQ1	12	2	1	1	1	40	24	3	no
SEC53	16	2	2	2	0	52	3	4	yes
SPT3	17	13	5	5	3	80	3	1	no
SSU1	29	5	9	5	7	96	25	3	no
STE2	19	18	7	8	5	100	18	4	no
SUP35	9	7	11	2	4	71	23	4	yes
TAO3	13	46	18	16	6	499	83	1	yes
TRP1	19	3	3	2	2	53	19	3	no
URA1	15	15	1	8	1	57	7	4	no
URE2	26	7	2	2	2	62	4	3	no
VMA1	9	47	14	44	12	65	3	4	no
YAR023C	17	5	11	4	8	25	36	1	no
YCR007C	16	5	3	2	1	49	48	1	no
YHL044W	19	7	16	4	5	52	75	1	no

ZDS2	59	8	5	7	4	44	28	1	no

1. Quantile 1: lowest 25% expression; Quantile 2: 25-50%; Quantile 3: 50-75%; Quantile 4: highest 25% expression.

Additional Results (see Results & Discussion; Fig. 2)

The analysis presented in the main paper, using ratios of nonsynonymous to synonymous polymorphism, will be conservative if synonymous sites are more likely to be constrained in highly expressed genes relative to low-expressed genes (as seems likely; see Results & Discussion); the proportion of the gene that is functionally unconstrained will be overestimated in highly expressed genes. Another way to test for elevated nonsynonymous polymorphism across gene expression categories is to analyze nucleotide diversity per site (theta). Estimates of theta per nonsynonymous site decline as gene expression increases (Fig. S1), yielding the same result as presented in the main paper.

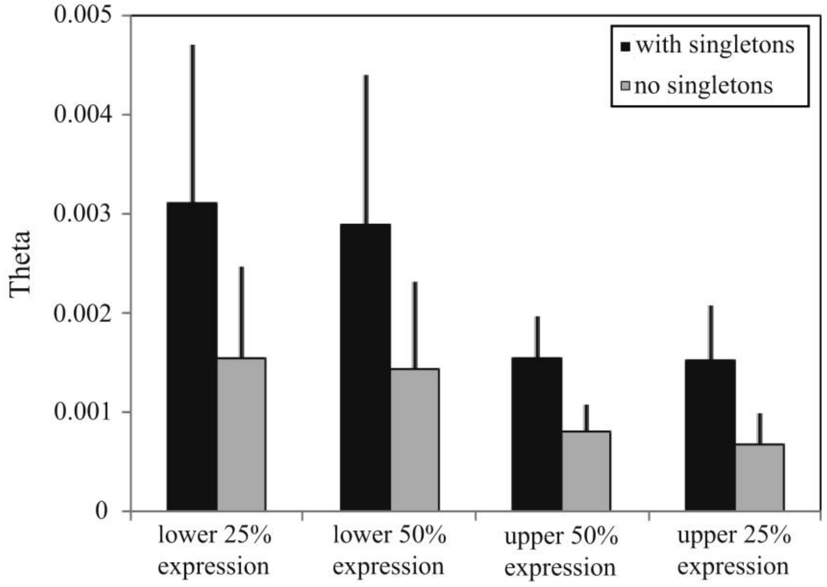

Fig. S1.— Nonsynonymous nucleotide variation (theta, per nucleotide site) as a function of gene expression. Mean theta and 90% confidence intervals are shown. See Fig. 2 legend for details about gene expression partitions. Highly expressed genes exhibit reduced levels of replacement variation: upper vs. lower 50% with singletons, $P = 0.040$; upper vs. lower 25% with singletons, $P = 0.069$; upper vs. lower 50% without singletons, $P = 0.084$; upper vs. lower 25% without singletons, $P = 0.067$; all t-tests are two-tailed.

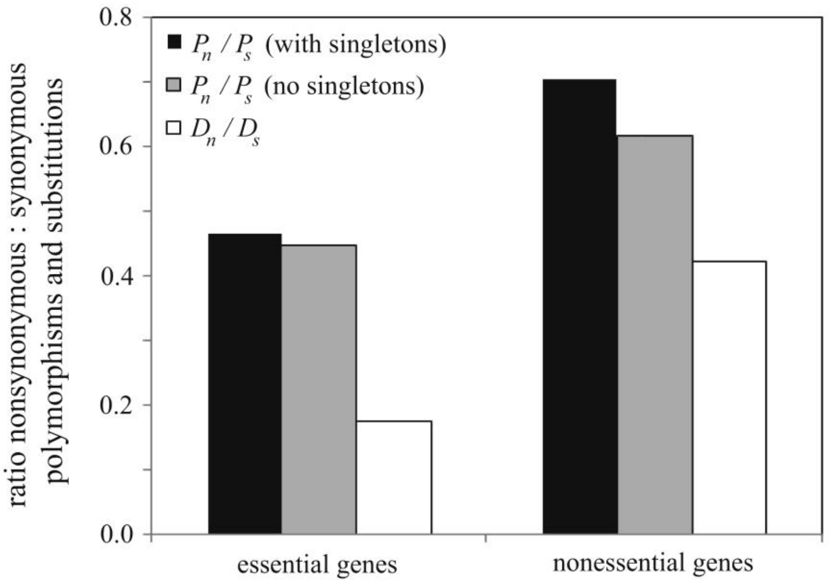

Fig. S2.— Ratios of replacement to silent polymorphism (P_n/P_s) in *S. cerevisiae*, and substitutions (D_n/D_s) between *S. cerevisiae* and *S. paradoxus*; genes are partitioned according to knockout viability phenotype. Results were obtained by pooling polymorphism and divergence data for multiple genes within each of the two categories. P_n/P_s ratios are lower in essential genes: with singletons, $P = 0.067$; without singletons, $P = 0.806$. D_n/D_s ratios are lower for essential genes ($P < 0.00001$).

II. Additional Figures

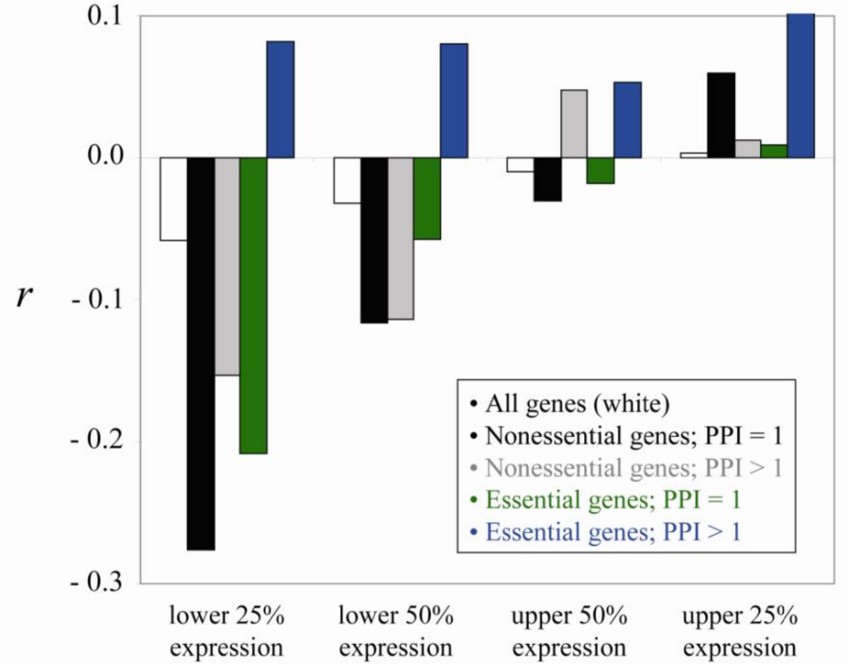

Fig. S3.— The relationship (*r* = partial correlation coefficient; see materials and methods) between recombination rate and *dN/dS* for four gene expression intervals.

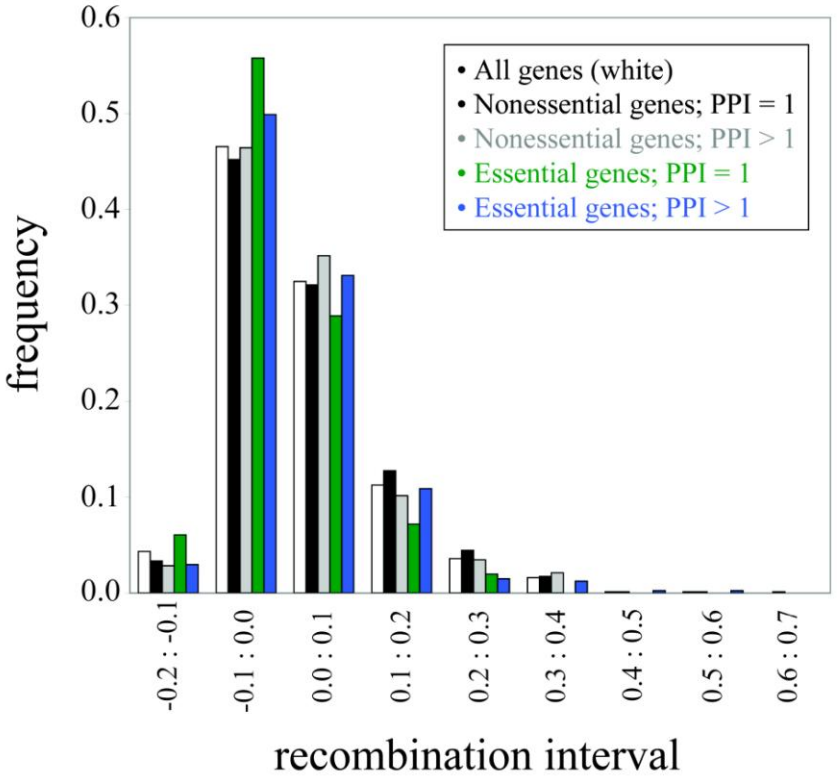

Figure S4.— The distribution of recombination rates for *S. cerevisiae* genes. Recombination rate per gene was estimated with a microarray experiment as described in Gerton et al. [8]. Recombination rates are plotted as the logarithm of the mean hybridization ratio of recombinant probes (*P2*) relative to a total genomic probe (*P1*); higher values of $\log_{10}$ (*P2*/*P1*) reflect higher frequencies of recombination.

III. Additional References

Smith NGC, Eyre-Walker A: Adaptive protein evolution in Drosophila. *Nature*, 2002 415:1022-1024.

Watterson EA: On the number of segregating sites in genetical models without recombination. *Theor Popul Biol*, 1975 7:256-276.
